# Transmission in Heteronymous Spinal Pathways Is Modified after Stroke and Related to Motor Incoordination

**DOI:** 10.1371/journal.pone.0004123

**Published:** 2009-01-05

**Authors:** Joseph-Omer Dyer, Eric Maupas, Sibele de Andrade Melo, Daniel Bourbonnais, Jean Fleury, Robert Forget

**Affiliations:** 1 Centre de recherche interdisciplinaire en réadaptation, Institut de réadaptation de Montréal, Montréal, Québec, Canada; 2 École de réadaptation, Faculté de médecine, Université de Montréal, Montréal, Québec, Canada; 3 Centre Mutualiste de Rééducation Fonctionnelle, Albi, France; 4 Université Paul Sabatier, Toulouse III, Toulouse, France; Emory University, United States of America

## Abstract

Changes in reflex spinal pathways after stroke have been shown to affect motor activity in agonist and antagonist muscles acting at the same joint. However, only a few studies have evaluated the heteronymous reflex pathways modulating motoneuronal activity at different joints. This study investigates whether there are changes in the spinal facilitatory and inhibitory pathways linking knee to ankle extensors and if such changes may be related to motor deficits after stroke. The early facilitation and later inhibition of soleus H reflex evoked by the stimulation of femoral nerve at 2 times the motor threshold of the quadriceps were assessed in 15 healthy participants and on the paretic and the non-paretic sides of 15 stroke participants. The relationships between this reflex modulation and the levels of motor recovery, coordination and spasticity were then studied. Results show a significant (Mann-Whitney U; P<0.05) increase in both the peak amplitude (mean±SEM: 80±22% enhancement of the control H reflex) and duration (4.2±0.5 ms) of the facilitation on the paretic side of the stroke individuals compared to their non-paretic side (36±6% and 2.9±0.4 ms) and to the values of the control subjects (33±4% and 2.8±0.4 ms, respectively). Moreover, the later strong inhibition observed in all control subjects was decreased in the stroke subjects. Both the peak amplitude and the duration of the increased facilitation were inversely correlated (Spearman r = −0.65; P = 0.009 and r = −0.67; P = 0.007, respectively) with the level of coordination (LEMOCOT) of the paretic leg. Duration of this facilitation was also correlated (r = −0.58, P = 0.024) with the level of motor recovery (CMSA). These results confirm changes in transmission in heteronymous spinal pathways that are related to motor deficits after stroke.

## Introduction

Basic sensorimotor mechanisms can regulate the activity of motoneurones (MNs) through different spinal pathways at segmental and intersegmental spinal cord levels. The implication of such pathways in the sensorimotor impairments following stroke is still unclear. Among these impairments, spasticity [1] and coordination deficits [2] are often observed in the paretic limb. Some investigators have studied the relationship between abnormal reflex modulation and sensorimotor impairments. For example, it has been shown that malfunction of the spinal pathways regulating the stretch reflex may contribute to the hypertonicity and hyperreflexia that characterize spasticity [3], [4]. However, very few studies have investigated the spinal mechanisms regulating the modulation of activity between muscles acting at different joints. Moreover, the relationships between this modulation and the coordination deficits that are often characterized by abnormal muscle synergies [2] have not been studied after stroke.

Changes in many segmental inhibitory circuits regulating inputs to MNs at the same spinal level and controlling muscles acting at a single joint have been reported following stroke. A decrease in the inhibition by Ib afferents [5] and of the presynaptic inhibition of Ia afferents [6], [7] have been observed. Abnormalities in reciprocal inhibition by Ia afferents [8], [9], [10], [11] and a malfunction of recurrent inhibition [12] have also been described. Some evidence suggests that the malfunction of these circuits may contribute to motor deficits of the paretic limb. Thus, a decrease of reciprocal inhibition has been related to changes in muscle tone in the paretic arm [10] and to hyperreflexia in the paretic leg [9]. Deficits in recurrent inhibition have also been related to spasticity in some stroke patients [13]. Furthermore, the reestablishment of reciprocal inhibition has been related to the motor recovery of the paretic leg [14]. Although the impairment of several segmental pathways is well documented and may explain agonist-antagonist relationships at the same joint, only few studies have investigated intersegmental pathways and their potential implication in the motor deficits following a stroke.

Intersegmental and propriospinal pathways participate in basic sensorimotor processes that influence MNs at different segmental levels and consequently muscles acting at different joints [15]. In humans, these pathways are explored with electrophysiological methods where a conditioning stimulus is used to modulate the reflex and voluntary activities of a heteronymous muscle [16], [17], [18]. For example, projections of group Ia pretibial flexors afferents to quadriceps (Quads) have been assessed by using the heteronymous facilitation of Quads reflex and voluntary firing of motor units after the stimulation of the common peroneal nerve [18], [19]. These pathways are under supraspinal and peripheral control [15], [16] and their malfunction secondary to the alteration of supraspinal influences following stroke could contribute to motor deficits of the paretic limb [20]. Recent investigations have shown an abnormal increase in the excitatory heteronymous influences of non-monosynaptic group I and group II proprioceptive afferents projecting from the common peroneal nerve to Quads in hemiparetic individuals [21], [22]. The question arises whether the impairment of other intersegmental pathways may contribute to motor deficits of the paretic leg.

The study of the intersegmental pathways linking Quads to soleus (Sol) is relevant in stroke because of the pathological extension synergy [23], the abnormal coactivation pattern [24], [25], [26] and the spasticity [27], [28] often observed in knee and ankle extensors of the paretic leg. Excitatory and inhibitory intersegmental projections linking Quads to Sol can be assessed by measuring the effects of femoral nerve (FN) stimulation on Sol reflex activity and on Sol EMG during voluntary contraction [29], [30]. Up to now, changes in the excitability of such pathways have not been documented after stroke. However, these pathways have been studied in healthy subjects. More precisely, FN stimulation induces a short-latency, short-duration facilitation of Sol reflex activity. The facilitation is immediately followed by a short-latency and longer lasting inhibition [29], [30]. Moreover, this heteronymous inhibition has been found to be modulated according to the requirement of postural tasks such as sitting and standing [30].

This study aims (1) to determine whether the heteronymous facilitation and inhibition of Sol reflex activity by FN stimulation are modified in hemiparesis following stroke and (2) if possible changes in these heteronymous modulations are related to the levels of motor deficits of the paretic leg.

## Materials and Methods

### Participants

Fifteen stroke individuals with chronic hemiparesis (mean±SD: 52±15 years; 6 females; 9 males) and fifteen healthy individuals (51±16 years; 8 females; 7 males) of similar age (Mann-Whitney U: P = 0.95) participated in the study. All participants gave their written informed consent to the study, which had been approved by the Institut de réadaptation de Montréal ethics committee. They were recruited based on the following inclusion criteria: a single cerebrovascular accident involving the motor cortex, internal capsule or sub-cortical areas as documented by brain imagery and resulting in motor deficits of abrupt onset affecting the contralateral leg. Moreover, all participants tested had detectable patellar and Achilles tendon reflexes in the paretic leg. Individuals with stroke were excluded if they were on antispastic drugs or anxiolytic or antidepressant medication at the time of the study, or if they had receptive aphasia, hemispatial neglect or passive range of motion limitation of the paretic leg that could interfere with the experimental positioning. Moreover, participants with orthopaedic or neurological disorders other than stroke or those with stimulators (e.g. pacemaker) or metallic implants were excluded. Demographic data for the individuals with stroke is presented in Table 1 together with scores for the clinical measurements of coordination, spasticity and motor recovery of the lower limb that were assessed as described below.

**Table 1:** Demographic and clinical data for participants with stroke.

Participant	Sex/age	Side of stroke:	Time since stroke (months)	CMSA Leg (/7)	CMSA Foot (/7)	LEMOCOT	CSI (/16)
1	F/57	R	110	4	4	2	7
2	F/46	L	67	5	6	26	8
3	M/47	L	93	6	7	20	8
4	F/31	L	116	5	4	14	12
5	M/68	R	50	3	2	2	9
6	M/75	R	103	5	4	13	6
7	M/27	R	126	5	3	5	14
8	F/33	L	109	6	5	10	13
9	M/60	R	93	6	5	31	8
10	M/47	L	58	5	4	3	7
11	F/45	L	168	5	3	19	11
12	M/57	L	173	3	2	1	9
13	F/62	R	63	6	7	19	9
14	M/56	R	72	4	4	1	10
15	M/75	R	53	5	5	8	8

**M** = male, **F** = female, **L** = left, **R** = right, **LEMOCOT** = Lower Extremity MOtor COordination Test; **CMSA Leg & CMSA Foot** = Chedoke-McMaster Stroke Assessment at the leg and foot; **CSI** = Composite Spasticity Index.

### Clinical assessment

Prior to the experimental sessions, participants with stroke were evaluated to document levels of motor recovery, coordination and spasticity at the paretic leg. Motor recovery was measured using the reliable Chedoke-McMaster Stroke Assessment (CMSA) subscales at the paretic leg (CMSA Leg) and foot (CMSA Foot) [31]. These subscales range from 1 (no residual motor function) to 7 (no residual motor impairment) and are based on Brunnstrom's stages of motor recovery of the lower extremity [23]. Coordination was measured using the Lower Extremity MOtor COordination Test (LEMOCOT), validated for individuals with stroke [32]. In this test, the participants are seated and instructed to alternately touch with their foot, as fast and as accurately as possible; two standardized targets placed 30 cm apart on the floor, in a 20-second period. The degree of spasticity of the paretic ankle was measured with a reliable composite spasticity index (CSI) designed for individuals with stroke. Practical considerations in the use of CSI are described in Levin and Hui-Chan (1993) [33]. Briefly, this index is a 16-point scale that includes subscales measuring the amplitude of the Achilles tendon tap jerk (4-point), duration of the clonus (4-point) and the resistance to passive stretching of ankle extensors at moderate speed (8-point). Interval values of 1–5, 6–9, 10–12 and 13–16 correspond to absent, mild, moderate and severe spasticity, respectively [34].

### Electrophysiological investigation

Participants were comfortably seated in an adjustable reclining armchair with the hip flexed (80°), the knee flexed (10°) and the ankle slightly plantarflexed (10°). EMG activities were recorded by pre-amplified (10×) bipolar surface electrodes with 10 mm of inter-electrode distance (Delsys, Inc., Boston, MA). The recording electrodes were secured to the skin over the belly of soleus (just below the lateral gastrocnemius) and vastus lateralis (VL). EMG activities were first amplified (5000×), then filtered (30–1000 Hz) (*Grass*, model 12 A 5) and finally, digitalized at a sampling rate of 5 kHz. EMG signals from VL and Sol were displayed on an oscilloscope and were stored on computer for off-line analysis.

#### Soleus H reflex

The soleus H reflex was obtained by the stimulation (1-ms duration monophasic rectangular pulse, 0.25 Hz) of the posterior tibial nerve with the active electrode placed in the popliteal fossa. The intensity of the test stimulation was progressively increased to obtain the thresholds and the maximal peak-to-peak amplitudes of the M wave and H reflex responses. H max/M max and H/M threshold intensity ratios were determined at the tested side of control individuals and at the paretic and non-paretic sides of individuals with stroke.

#### Conditioning stimulation of femoral nerve

The FN was stimulated percutaneously at the femoral triangle (just lateral to the femoral artery) with a 1-ms duration monophasic rectangular pulse delivered by a cathode (half-ball of 2-cm diameter). The anode (11.5 cm×8 cm) was placed at the postero-lateral aspect of the buttock. The intensity of stimulation was progressively increased to obtain H reflex and M response thresholds of VL. The experiment was performed at an intensity of 2×MT (threshold of the M wave of VL) on one side (randomly chosen) of control participants and on the paretic and the non-paretic sides of those with stroke. In some subjects, the skin next to the FN was stimulated at the same intensity in order to produce the same sensation but without directly stimulating the nerve.

In this experiment, it must be considered that the distances travelled by the conditioning (from FN) and the test (from tibial posterior nerve) stimulations are unequal. The stimulation of the tibial nerve (test) must be delivered ahead of the conditioning stimulation of FN (negative conditioning-test intervals) for the simultaneous arrival of the two volleys on soleus MNs, which corresponds therefore to zero central delay. This delay was estimated from the latency of soleus H reflex and from the difference in afferent conduction times of conditioning (more proximal) and test volleys [30], [35]. It has been shown that the synchronous arrival of Ia volleys from FN and tibial posterior nerve occurs at interstimulus intervals (ISI) of about −5 to −7 ms [36]. Thus, the modulation of Sol H reflex induced by FN was assessed at ISIs that varied randomly from −10 ms to 40 ms (with increasing ISI steps of 0.5 ms from −10 to 1 ms ISI and steps of 5 ms from 1 to 40 ms ISI). For a given ISI, 12 unconditioned (control) and 12 conditioned reflexes (test) were delivered randomly. The amplitude of the unconditioned H reflex was kept at about 20% of Mmax during the test. The first tested side (paretic vs. non-paretic) was also randomly determined for each individual with stroke.

### Data analysis

Conditioned H reflexes of Sol were measured peak-to-peak. Modulation was calculated as the difference between the conditioned and unconditioned H reflex and expressed as a percentage of the unconditioned (control) H reflex. For each experiment, the size of the peak facilitation and of the maximal inhibition were measured as the largest amount of increase and the largest amount of decrease of the control reflex observed respectively within the time course from −10 ms to 40 ms ISI. In order to determine whether there were significant facilitation and inhibition from pre-onset ISI (i.e. before the beginning of the facilitation), analysis of variance (ANOVA) using Scheffe's method was performed. The onset and the end of the facilitation were determined as the first and last ISI at which the conditioned reflex was respectively larger and smaller than 5% of the control reflex. The duration of the facilitation was simply measured as the difference between the last and first ISI at which the facilitation occurs. The Wilcoxon signed-rank test was also used to compare the values of H reflex amplitudes between the paretic and non-paretic sides in the group with stroke. The Mann-Whitney U test was used to compare the values obtained between the two groups (control vs. stroke). The correlation between clinical scores (LEMOCOT, CMSA, CSI) and electrophysiological data was obtained using the Spearman rank test.

## Results

### Modulation of soleus H reflex across participants

**Figure 1:**
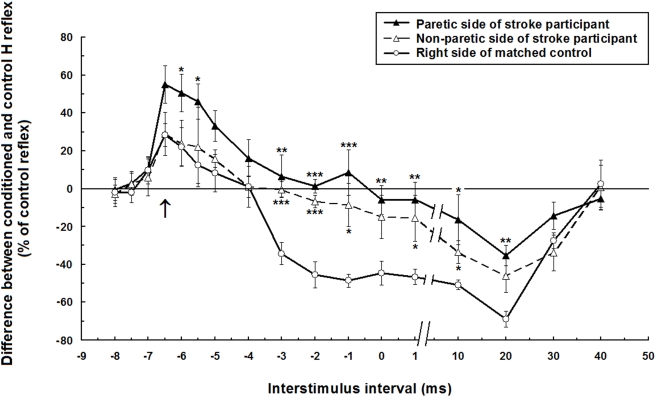
Time course of the heteronymous modulation Modulation of the H reflex evoked by stimulation of the femoral nerve at two times motor threshold of vastus lateralis. The difference between the conditioned and control H reflex (expressed as a % of the control reflex) is plotted against the conditioning-test interval. Data are from a stroke participant (#2, Table 1) and a matched control that showed similar ISIs for the onset (−7 ms) and the peak amplitude (−6.5 ms) of the facilitation and for the maximal inhibition (20 ms). Each symbol represents the mean of 12 measurements. Vertical bars = 1 SEM. Arrow = estimated time of simultaneous arrival of conditioning (from femoral nerve) and test (from tibial posterior nerve) volleys at the motoneurone level of soleus. Asterisks represent significant difference between modulation of the stroke participant (on the paretic side or the non-paretic side) and the right side of the matched control (* p≤0.05; ** p≤0.01; *** p≤0.001).

The results showed an increase in the early facilitation and a decrease in the later inhibition of Sol H reflex by the heteronymous influences from Quads on the paretic side of participants with stroke. Figure 1 presents the time course of the modulation of Sol H reflex amplitude evoked by the stimulation of femoral nerve in the right leg of a control and in the paretic and non-paretic legs of a participant with stroke (#2, Table 1). As previously described in healthy individuals, the modulation consists in an early and short-lasting phase of facilitation and a later longer-lasting phase of inhibition. Comparison is drawn here between these two participants who presented an early facilitation at similar latencies of onset (−7 ms ISI) and peak amplitude (−6.5 ms ISI). The ISI of maximal inhibition (20 ms) was also similar in these participants. At −6 ms ISI, facilitation at the paretic leg (51% increase compared to the unconditioned control reflex) was significantly greater (P<0.05) than facilitation in the non-paretic leg (24%) and than in the tested leg of the control subject (22%). A later phase of sustained inhibition from −3 ms to 30 ms ISI was observed in the control participant whereas this inhibition started later and was reduced in the paretic leg. A significant decreased inhibition was also observed on the non-paretic side but to a lesser extent than on the paretic side.

**Figure 2:**
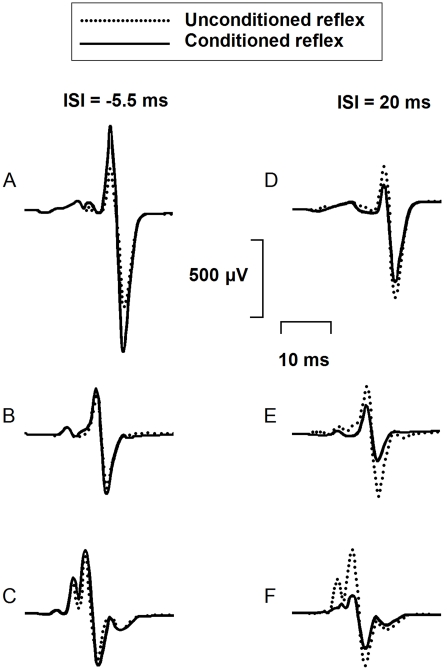
Effects of femoral nerve stimulation on soleus H reflex Averaged soleus EMG responses on the paretic side (A, D) and on the non-paretic side (B, E) of a stroke participant (#10, Table 1) and on the right side of a control participant (C, F) are presented at two different ISIs (−5.5 ms on the left side of the figure and 20 ms on the right side). Dotted lines are control H reflex (without FN stimulation), continuous lines are conditioned H reflex. Each trace represents the average of twelve EMG responses. Size of the control H reflex was at 20% of the size of the maximum M response in both participants.

Figure 2 presents means of twelve unconditioned H reflexes (dotted line) and twelve conditioned H reflexes (continuous line) in another participant with stroke (#10, Table 1) and another control participant. Results are shown at the ISI of peak facilitation (ISI = −5.5 ms; A, B, C on the left side of the figure) and at the ISI of maximal inhibition (ISI = 20 ms; D, E, F on the right) for both participants. At −5.5 ms ISI, facilitation of the H reflex (61% of the control reflex) was larger on the paretic side (A) than facilitation on the non-paretic side (16%)(B), and than on the right side of the control participant (12%)(C). At 20 ms ISI, inhibition of the reflex (27% decrease of the control reflex) on the paretic side (D) was less than on the non-paretic side (50%) (E), and than on the right side of the control (53%)(F).

A significant (P<0.05; Scheffe's method) facilitation was observed in 73% of control participants (11/15), in 80% of those with stroke on the paretic side (12/15) and in 67% of them on the non-paretic side (10/15). Moreover, peak facilitation was significantly larger on the paretic leg than on the non-paretic leg in 67% of participants with stroke (10/15). The inhibition was significant in all of the control participants and on the non-paretic side in all of those with stroke. However, it was absent on the paretic side in 20% of participants with stroke (3/15). The maximal amount of inhibition was also significantly smaller (i.e. less inhibition) on the paretic than on the non-paretic leg in 40% of them (6/15). Stimulation of the skin next to the nerve always failed to produce modulation of the soleus H reflex in all of the experiments in which it was performed.

### Modulation of soleus H reflex across groups

**Figure 3:**
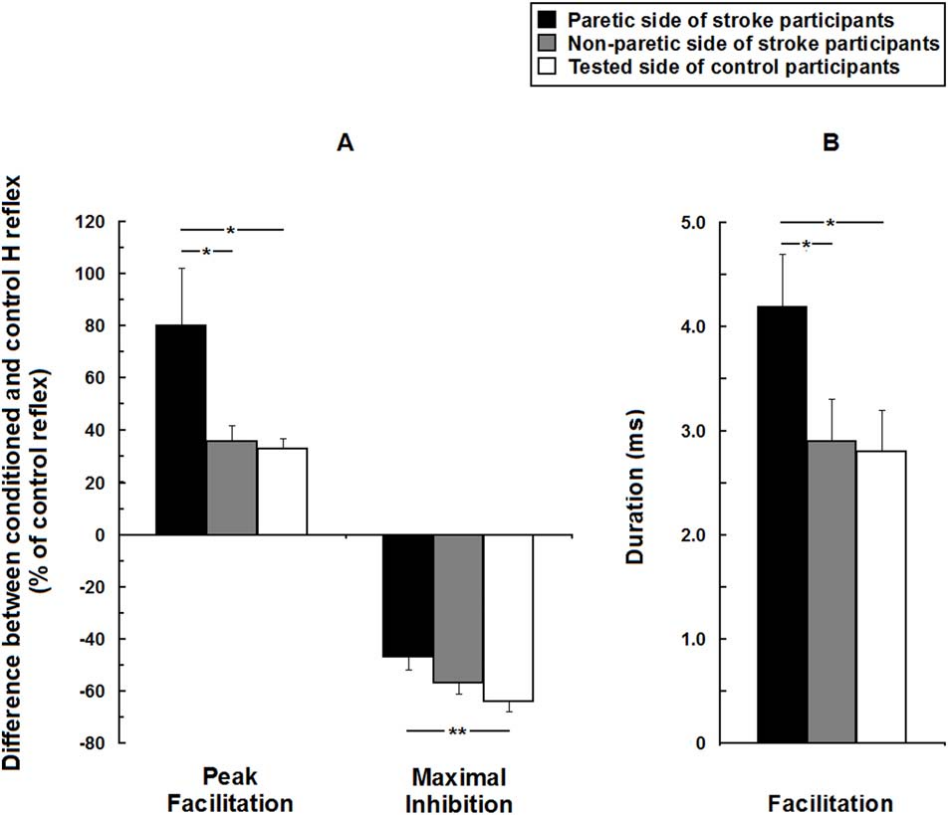
Group comparisons of the heteronymous modulation of soleus H reflex in 15 hemiparetic and 15 healthy participants Mean peak facilitation and maximal inhibition of soleus H reflex induced by the stimulation of femoral nerve (expressed as a % of the control reflex) observed from −10 to 40 ms ISI (A). Mean duration of the facilitation calculated as the difference between the latest and the earliest ISIs at which it was observed (B). Vertical bars = 1 SEM. Asterisks represent significant difference in modulation between the paretic side, the non-paretic side of stroke participants and the tested side of control participants (* p≤0.05; ** p≤0.01).

Since peak facilitation and maximal inhibition are not necessarily achieved at exactly the same ISI across subjects, Figure 3 presents the mean peak facilitation and the maximal inhibition in the two groups, independently of the ISI (A). The mean duration of the early facilitation is also presented for the two groups (B). Both the peak and the duration of the facilitation were larger on the paretic side (mean±SEM: 80±22% and 4.2±0.5 ms respectively) than on the non-paretic side (36±6% and 2.9±0.4 ms) of those with stroke and than on the tested leg of control participants (33±4% and 2.8±0.4 ms). The mean maximal inhibition measured on the paretic side of participants with stroke (decrease of 47±5% of the control reflex) was not significantly different from that on the non-paretic side (57±4%) but it was smaller (i.e. less inhibition) than that of the control group (64±4%).

**Figure 4:**
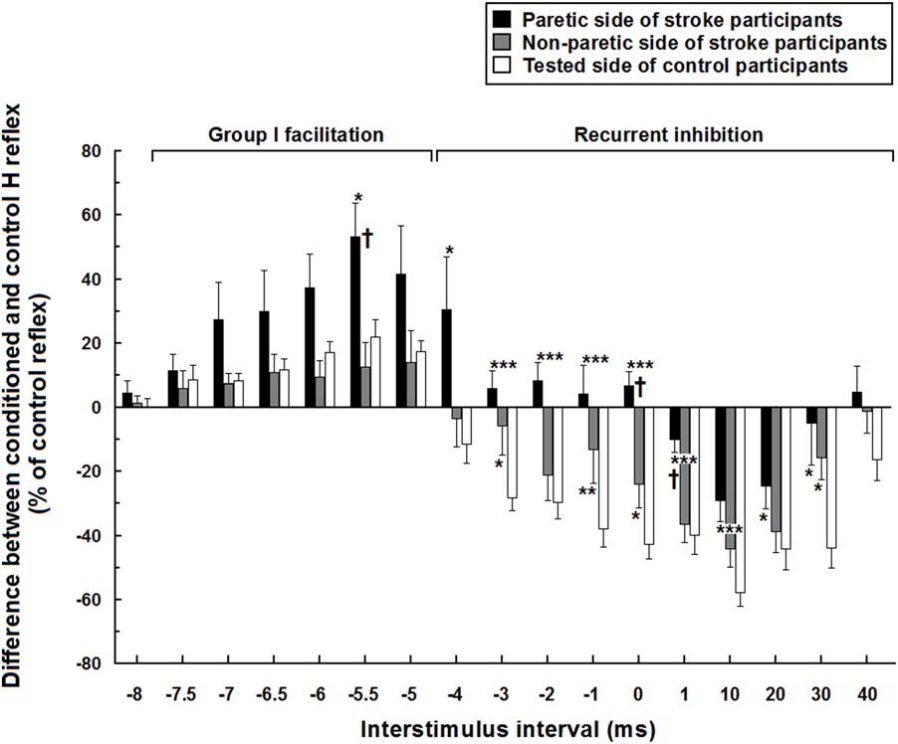
Mean time course of soleus H reflex modulation in 15 hemiparetic and 15 healthy participants Mean modulations are expressed in % of the control H reflex and plotted against the different interstimulus intervals (ms) between femoral nerve stimulation and posterior tibial nerve stimulation. Vertical bars = 1 SEM. Asterisks represent significant difference in modulation between the paretic side or non-paretic side of stroke participants and the tested side of control participants (* p≤0.05; ** p≤0.01; *** p≤0.001). Crosses represent significant difference between the paretic and non-paretic sides of stroke participants († p≤0.05).

Figure 4 presents the full time course of the mean modulation in both groups at different ISI. The mean facilitation reached its peak at −5.5 ms ISI and was significantly larger on the paretic side (53±11%) than on the non-paretic side (13±8%) of participants with stroke and than on the tested leg (22±5%) of the control participants. There were significant differences in the mean modulations observed from −4 ms to 30 ms ISI, when the paretic leg was compared to the non-paretic leg or to the tested leg in control participants. The mean inhibition observed from −4 ms to 40 ms ISI in controls was replaced by facilitation from −4 ms to 0 ms ISI and was markedly reduced from 1 ms to 30 ms ISI on the paretic leg. The inhibition was also altered on the non-paretic side but less than on the paretic side. For example, there was significantly (Mann-Whitney U; P<0.05) less inhibition on the non-paretic side at the −3 ms, −1 ms and 30 ms ISIs (6±9%, 13±11% and 16±7%, respectively) compared to that of control participants (29±4%, 39±6% and 45±6%)

### Excitability of the soleus H reflex

The mean soleus Hmax/Mmax ratio was higher (Wilcoxon: P<0.05) on the paretic side of participants with stroke (mean±SD: 0.80±0.19) than on their non-paretic side (0.57±0.26) and than on the tested leg of control participants (0.55±0.36) (Mann-Whitney U: P<0.05). The mean soleus H/M thresholds ratio on the paretic leg of those with stroke (mean±SD: 0.87±0.20) was significantly lower (Mann-Whitney U; p<0.05) than on the tested leg of the controls (0.99±0.17). The mean H/M thresholds ratio on the non-paretic side (0.99±0.28) was similar to that of control participants but was not significantly different from that measured on the paretic side due to a large variability.

### Clinical correlations

The coordination score of the paretic lower limb, as assessed using the LEMOCOT scale, was correlated with the motor recovery score of the leg (CMSA Leg) (Spearman r = 0.79; P<0.001) and of the foot (CMSA Foot) (r = 0.66; P = 0.008) but not with the spasticity index (CSI). The LEMOCOT score of the paretic lower limb was inversely correlated with the level of the peak facilitation (Spearman r = −0.65; P = 0.009) and the duration of the facilitation phase (r = −0.67; P = 0.007). There was also correlation between the level of motor recovery of the paretic leg (CMSA Leg) and the duration of the facilitation phase (r = −0.58; P = 0.024). The level of motor recovery of the paretic foot (CMSA Foot) and the degree of spasticity (CSI) were not correlated with the characteristics of the facilitation. No correlations were found between the clinical scores of the paretic lower limb (LEMOCOT, CMSA Leg, CMSA Foot, CSI) and the level of the later inhibition phase.

## Discussion

The heteronymous modulation of soleus H reflex induced by the stimulation of FN is modified in individuals with stroke. Moreover, increases in the level and duration of the early facilitation are inversely correlated with the coordination score (i.e. the higher the amplitude and duration of the facilitation, the lower the coordination score). An inverse correlation is also found between duration of facilitation and level of motor recovery of the paretic leg. Before commenting on these aspects, the characteristics of the modulation observed in the healthy individual will be discussed in the context of the neurophysiological mechanisms reported in the literature on humans.

### The modulation of soleus H reflex induced by FN stimulation

It has been shown that stimulation of the femoral nerve evokes an early facilitation and a later inhibition of the soleus H reflex, and also of the poststimulus time histogram of single voluntarily activated motor units and of the ongoing EMG of soleus voluntarily activated [29], [35], [36]. It has been argued that the heteronymous facilitation of soleus H reflex is brought about by projections of group Ia afferents from FN to soleus motoneurones, because of their short latency (average onset at −6.5 ms ISI) [37] and low threshold of apparition (0.65×Quads motor threshold) [38]. Indeed, the ISI at which the facilitation begins during the reflex conditioning corresponds to the peripheral distance between the two stimulated sites with no central delay. Moreover, the short latency (about 33 ms) and low threshold (0.77×Quads MT) of the facilitation of soleus voluntarily activated single motor unit during PSTH experiments corresponds to the fastest and the lowest threshold reflex pathway between quadriceps and soleus [35], [39]. Thus, the latency of the facilitation of soleus activated voluntarily corresponds to the synchronous arrival of group Ia afferents evoked by the conditioning (femoral nerve) and testing (tibial posterior nerve) stimuli. The monosynaptic origin for the first 0.5 millisecond of this facilitation has been previously studied in cats and humans and its measurement, as discussed below, has been proposed as a method to assess presynaptic inhibition of Ia afferents [38], [40].

The threshold of activation, the latency and the duration of the later inhibition phenomenon strongly suggest the participation of a recurrent inhibition mechanism by Renshaw cells projecting from quadriceps to soleus motoneurones [35], [36]. Such intersegmental influence of Renshaw cells has also been suggested in the human upper limb [41]. In our paradigm, the activation of quadriceps motoneurones would result in the firing of its Renshaw cells inhibiting soleus motoneurones.

The afferent volley produced by the conditioning stimulation of FN (at 2×MT) in our study could activate other pathways such as Ib non-reciprocal inhibition [42] and those activated by cutaneous afferents. However, the contribution of those afferents is probably limited considering: a) the intensity of the stimulation needed to evoke the inhibition of soleus reflex and b) the duration of the response. In fact, the threshold for Ib activation is around 0.6×MT [17] whereas the observed heteronymous inhibition of soleus appears above 1×MT [35]. Moreover, Ib inhibition evoked by a single volley has a duration of less than 10 ms in humans [43] and thus could influence only the beginning of the observed inhibition of about 40 ms duration. On the other hand, the possibility has been raised that the Ib afferent discharge produced by either the quadriceps twitch contraction or the soleus reflex contraction contributes to the later inhibition [44].

Evidence also suggests that the stimulation of cutaneous afferents is not sufficient to produce heteronymous modulation in this pathway in humans. Bergmans et al. (1978) have shown that a conditioning stimulus with large plate electrodes applied to the internal saphenous nerve (a purely cutaneous branch of the femoral nerve) failed to produce the short-latency facilitation of soleus [36]. Other studies have shown that pure cutaneous stimulation failed to evoke the later heteronymous inhibition of soleus [39], [45]. In our participants, moving the electrode off the nerve and stimulating the skin to produce the same sensation failed to produce a modulation of the soleus H reflex.

### Mechanisms underlying modulation changes in the paretic leg

The changes in the modulation of soleus H reflex found in those with stroke may result from 1) a decrease of the presynaptic inhibition of quadriceps Ia terminals projecting onto soleus motoneurones; 2) an increase in the transmission of non-monosynaptic group I and II facilitation; 3) an increase in the excitability of soleus α motoneurones and 4) a decrease in recurrent intersegmental pathways.

Presynaptic inhibition (PI) of quadriceps Ia afferents projecting to soleus can be explored by assessing the changes within the first 0.5 ms of the facilitation of soleus H reflex induced by FN stimulation [40], [46]. Using this method, PI has been reported to be normal in the paretic leg, since no increase in the early component of the heteronymous facilitation has been measured [46]. This is in accordance with our results, which showed no significant difference within the first 0.5 ms after the onset of the early facilitation in the paretic leg. However, the facilitation was increased in its early phase on the paretic leg of some participants with stroke but this phenomenon becomes significant only 2 ms after the onset of facilitation. This implies that although a change in PI cannot be ignored, it might not be the primary mechanism responsible for the increased facilitation. The malfunction of PI in hemiparesis is not clearly established and the interpretation of the studies depends on the method of exploration of this mechanism and the spinal level tested. When using the vibratory-induced inhibition of reflex activity, PI has been found to be reduced in the paretic leg [7] and this decrease may play a role in spasticity [47]. However, the precise neurophysiological mechanism underlying the vibration-induced inhibition is not fully understood and a post-activation depression in neurotransmitter release rather than PI could be involved [46], [48], [49]. Another method to assess PI is with the measurement of the presynaptic inhibition phase of reciprocal disynaptic inhibition elicited by a stimulation applied to the nerve supplying antagonistic muscles. Using this method, PI has been found to be impaired in the paretic upper limb [8]. With respect to the paretic lower limb, contradictory results have been obtained since a decreased [14] as well as a normal PI [6] have been reported at rest, whereas an alteration of the modulation of PI during gait has been suggested [50].

Other mechanisms may contribute to the facilitation of transmission in the pathways mediating the heteronymous excitation of soleus reflex activity by quadriceps afferents in the paretic leg. The increase of facilitation from 2 ms to 7.5 ms after its onset (i.e. −5.5 ms to 0 ms ISI) may involve the malfunction of oligosynaptic intersegmental excitatory influences from quadriceps afferents. Propriospinal systems of interneurones are known to have polysynaptic intersegmental excitatory influences on heteronymous muscles in the human lower limb [18], [51], [52]. The malfunction of such short propriospinal-like interneurones interposed between quadriceps and soleus spinal segments in hemiparesis could result in an improper regulation of the polysynaptic influences of group I afferents from FN to soleus MNs. This could also affect, at least theoretically, transmission in group II intersegmental excitatory influence. However, such group II influence has been observed from plantar foot muscles to human leg and thigh motoneurones but not from femoral nerve to soleus MNs [53], [54]. Facilitation of transmission in heteronymous pathways has been demonstrated in the paretic leg of individuals with stroke. An increased facilitation of quadriceps reflex activity by group I non-monosynaptic and group II afferents from the common peroneal nerve has been observed in the paretic leg and this change was not correlated with the spasticity level measured with the Ashworth scale [21], [22]. These authors have proposed that an increase in the excitability of the relevant lumbar premotor neurons resulting from a disruption of the excitation/inhibition balance by their supraspinal control might account for the increased excitation by group I and II afferents [22].

The higher Hmax/Mmax and lower H/M threshold ratios of the soleus H reflex in the paretic leg would reflect an increase in the excitability of soleus α motoneurones if PI is not impaired in the lower limb. Such increase in the excitability of soleus motoneurones may contribute to the increase of facilitation and mask the effects of the later recurrent inhibition mechanism. However, if the hyperexcitability of soleus motoneurones is involved, it is not the main mechanism responsible for the changes in the intersegmental pathways in the paretic leg since no correlation was found between the H/M ratios and the heteronymous reflex modulation.

Assuming that the heteronymous inhibition is of Renshaw origin [35], our results are compatible with a reduction of recurrent inhibition of soleus MNs by quadriceps MNs discharge in the paretic leg. The early effect of recurrent inhibitory pathways may have been masked by an increase of group II excitatory influences on the paretic leg. The absence of evidence of group II excitation from quadriceps to soleus in healthy subjects may result from concomitant effects of the recurrent inhibition. In stroke individuals, the increase of group II excitation would probably affect mostly the first part of the inhibition since it has been demonstrated that group II afferents normally have their peak influence from 6 to 20 ms after the onset of the early group I induced facilitation [22], [53], [54], [55]. Our results suggest that recurrent inhibition could also be impaired in itself since the inhibition is reduced at the late ISIs (30 ms and 40 ms ISI) at which the influence of group II excitation must be diminished. Recurrent inhibition has previously been assessed using the method of conditioning soleus H reflex by a previous reflex discharge that activates Sol Renshaw cells [56]. Using this method, it has been found to be normal or increased at rest in hemiparesis [12], [57]. However, an abnormal modulation of recurrent inhibition of soleus has been observed during voluntary and postural contractions [12]. Simon (1996) first observed a supranormal level of activity of Renshaw cells associated with flaccidity in patients with stroke. Later however, at follow-up, a reduction of recurrent inhibition at rest, in parallel with the clinical development of spasticity, was revealed [13].

### Mechanisms underlying modulation changes in the non-paretic leg

The early increased facilitation found in the paretic leg of stroke participants was not observed on their non-paretic side. However, a significant reduction of the following inhibition was revealed compared to that of the control participants, although not as severe as on the paretic side. This reduced inhibition in the non-paretic leg reflects bilateral impairments in spinal pathways after unilateral stroke. Such malfunction in the non paretic leg could theoretically be explained by 1) an abnormal modulation of contralateral afferents from the paretic side, 2) an influence of the lesioned cerebral hemisphere on ipsilateral premotor interneurones and 3) changes in the non-lesioned cerebral hemisphere affecting the non-paretic side.

Bilateral influences of group II afferents have been demonstrated. Indeed, a large majority of midlumbar interneurones of the spinal cord of cats are activated by ipsilateral and contralateral stimulation of group II afferents, whereas similar crossed actions from group I afferents are rare [58]. Indirect evidence could also suggest a bilateral influence of group II afferents in humans. Unilateral displacement of the lower limb evokes bilateral EMG responses in leg and foot muscles in standing humans [59]. Medium-latency stretch responses of lower limb muscles in humans are thought to be transmitted to the spinal cord by secondary spindle afferents and relayed through an oligosynaptic spinal pathway [60], [61], [62], [63]. These contralateral responses are delayed on the spastic side of stroke individuals and it has been suggested that regulation of the relevant spinal interneurones is impaired because of malfunction of their supraspinal control after stroke [64]. In the human thumb, there is evidence that long latency stretch responses reflect a trans-motor-cortical long loop response and that corticospinal tract malfunction can modify contralateral muscle response after unilateral stretch [65].

Changes in the transmission of group II pathways in the non-paretic leg may also result from impairment of the ipsilateral supraspinal influence on premotor interneurones that receive input from group II afferents. This supraspinal ipsilateral influence has been demonstrated in cats [66]. Finally, several studies have found changes in the cerebral hemisphere contralateral to the cerebral lesion [67], [68], [69], [70], [71] and these changes can potentially affect the regulation of the spinal mechanisms involved in the control of the non-paretic side.

### Clinical correlations and functional considerations

The coordination score (LEMOCOT) of the paretic leg was correlated with the peak of the early facilitation and its duration when stimulating femoral nerve afferents at 2×MT. First, this suggests that motor impairment in the paretic leg is related to increased facilitation of transmission in these excitatory pathways. Second, that it could involve high threshold afferents such as oligosynaptic group I and group II afferents. Group II afferents threshold has been estimated to be reached at 2.1 times the threshold of the fastest conducting Ia afferents [54], which is estimated at 0.5 to 0.6×MT [18]. This is relevant since, as we mentioned earlier, the major response of stretching in a voluntarily activated muscle is a medium latency response that could be mediated by group II muscle afferents [61], [72]. Thus, malfunction of these pathways may have a functional impact.

The widespread heteronymous distributions of Ia excitation and recurrent inhibition in humans is thought to assist bipedal stance and gait [35], [73]. Since quadriceps and soleus are both anti-gravity muscles, with an out-of-phase reciprocal activation during gait, the coordination of their activity is relevant for human locomotion. Quadriceps reaches its peak of activation at the early stance phase in order to support body weight [74]. In turn, calf muscles show their maximal activity at the late stance phase to counteract the passive ankle dorsiflexion and facilitate push off [75]. The coordination of quadriceps and soleus reciprocal activity must be adequately regulated in order to achieve gait efficiency and intersegmental pathways are strategically positioned to participate in this coordination. The intersegmental pathways linking these two muscles are regulated according to their level of activation [76], [77], the task specificity [77] and the postural requirement [30]. Very few studies have investigated the possible relationship between impaired spinal mechanisms and the motor deficits observed in hemiparesis. Okuma and Lee (1996) have reported a deficit of disynaptic reciprocal inhibition between ankle flexors and extensors in hemiparetic individuals [14]. Moreover, an improvement (i.e. increase) in this mechanism has been correlated with decreased spasticity and increased strength of tibialis anterior in those with stroke.

In the present study, changes in the modulation between knee and ankle extensors were not significantly correlated with spasticity. The stronger and significant inverse correlation with coordination implies that the deficits observed could be factors that contribute more to incoordination than to spasticity. To our knowledge, correlation between muscle tone and hyperreflexia levels and changes in reflex modulation mechanisms have been reported only in homonymous or in antagonist muscles controlled by neural circuits of the same segmental level [9], [10]. The few other studies [21], [22], like our own, investigating intersegmental mechanisms modulating activity between heteronymous muscles at different joints in hemiparetic subjects did not find correlations between modulation changes and spasticity levels after stroke.

An increased intersegmental facilitation and decreased inhibition between quadriceps and soleus could result in increased abnormal coactivation of these muscles during functional tasks. This could possibly strengthen the abnormal extensor synergy so frequently observed at the lower limb in hemiparesis after stroke. Future studies should investigate the possible link between the integrity of intersegmental pathways and motor impairments of the paretic leg, particularly with regard to the incoordination reported in the affected paretic lower limb [24], [78], [79] and upper limb [80] during functional tasks.

### Conclusion

The facilitatory and inhibitory modulations of the soleus H reflex by FN stimulation are both impaired in hemiparesis following stroke; facilitation is increased and inhibition decreased. Based on previous studies, this suggests a malfunction of the excitatory Ia afferent projections and recurrent inhibition from the quadriceps to the soleus motoneurones, although additional mechanisms could be involved. The prolongation of the facilitation phase in individuals with stroke may potentially involve a facilitation of transmission in heteronymous group II pathways. Modulation of transmission in this pathway may even be affected on the non-paretic side. Moreover, the increased transmission was related to the level of impairment of coordination in the paretic leg. Such spinal malfunction may contribute to the motor deficits observed in the paretic leg and particularly in the incoordination of muscles acting at different joints. Future studies should examine if and how these faulty modulations influence abnormal locomotor patterns during functional tasks following stroke.
